# Degree of impaired kidney function at hospital discharge has a major impact on long-term survival of critically ill patients recovered from renal failure

**DOI:** 10.1186/cc10982

**Published:** 2012-03-20

**Authors:** S Stads, G Fortrie, J Van Bommel, R Zietse, M Betjes

**Affiliations:** 1 Erasmus MC, Rotterdam, the Netherlands

## Introduction

Renal replacement therapy (RRT) in critically ill patients with acute kidney injury (AKI) is associated with high mortality. However, little is known about the prognosis of renal function after ICU discharge and the effect of persisting impaired kidney function on long-term survival. The objective of this study was to evaluate the overall long-term mortality in a cohort of ICU patients with AKI necessitating RRT. We hypothesized that both patient characteristics and the degree of renal insufficiency at hospital discharge will influence long-term mortality.

## Methods

A retrospective cohort study was performed including all patients older than 18 years admitted to the ICU of a tertiary-care center between 1994 and 2010, who underwent continuous RRT during their ICU stay (*n *= 1,220).

## Results

In-hospital mortality was 54.9%. After hospital discharge, the overall mortality was 75.3% after a median follow-up of 8.5 years (range 1 to 17 years). Univariate analysis showed that age, surgical or nonsurgical reason for ICU admission and kidney function at discharge were associated with overall survival. Multivariate Cox regression analysis of the association of kidney function at hospital discharge with patient survival was performed, adjusting for age, sex and surgical or nonsurgical admission type. The eGFR at hospital discharge remained independently associated with long-term survival (*P *< 0.001). Only 87 (15.8%) patients were discharged with an eGFR >90 ml/minute (using the MDRD formula). In this group 5-year and 10-year survival were respectively 77.6% and 66.7%. The mortality risk increased for every increase in stage of chronic kidney disease (hazard ratio 1.25, *P *< 0.001). Patients discharged with an eGFR <30 ml/minute (CKD 4 to 5, 37.3% of patients at hospital discharge) had a 5-year and 10-year survival of only 42.5% and 28.5%.

## Conclusion

ICU patients with AKI who received CRRT have a high mortality risk. This is more outspoken for patients who experience incomplete recovery of renal function at hospital discharge. Impaired kidney function at discharge has a major negative impact on their long-term survival. These results stress the importance of preserving kidney function in ICU patients and the need for long-term nephrological follow-up. Future research will have to identify possible determinants in the period following hospital discharge that can be used to prolong survival in these patients.

